# Two Novel α7 Nicotinic Acetylcholine Receptor Ligands: In Vitro Properties and Their Efficacy in Collagen-Induced Arthritis in Mice

**DOI:** 10.1371/journal.pone.0116227

**Published:** 2015-01-24

**Authors:** Marjolein A. van Maanen, Roger L. Papke, Frieda A. Koopman, Jessica Koepke, Lisette Bevaart, Roger Clark, Diana Lamppu, Daniel Elbaum, Gregory J. LaRosa, Paul P. Tak, Margriet J. Vervoordeldonk

**Affiliations:** 1 Department of Clinical Immunology & Rheumatology, Academic Medical Center/University of Amsterdam, Amsterdam, The Netherlands; 2 Department of Pharmacology and Therapeutics, University of Florida College of Medicine, Gainesville, Florida, United States of America; 3 Arthrogen BV, Amsterdam, The Netherlands; 4 Cornerstone Therapeutics, Inc., Cary, North Carolina, United States of America; 5 DEC Associates, LLC, Newton, Massachusetts, United States of America; Faculté de médecine de Nantes, FRANCE

## Abstract

**Introduction:**

The cholinergic anti-inflammatory pathway can downregulate inflammation via the release of acetylcholine (ACh) by the vagus nerve. This neurotransmitter binds to the α7 subunit of nicotinic acetylcholine receptors (α7nAChR), expressed on macrophages and other immune cells. We tested the pharmacological and functional profile of two novel compounds, PMP-311 and PMP-072 and investigated their role in modulating collagen-induced arthritis (CIA) in mice.

**Methods:**

Both compounds were characterized with binding, electrophysiological, and pharmacokinetic studies. For in vivo efficacy studies in the CIA model the compounds were administered daily by oral gavage from day 20 till sacrifice at day 34. Disease progression was monitored by visual clinical scoring and measurement of paw swelling. Inflammation and joint destruction were examined by histology and radiology.

**Results:**

Treatment with PMP-311 was effective in preventing disease onset, reducing clinical signs of arthritis, and reducing synovial inflammation and bone destruction. PMP-072 also showed a trend in arthritis reduction at all concentrations tested. The data showed that while both compounds bind to α7nAChR with high affinity, PMP-311 acts like a classical agonist of ion channel activity, and PMP-072 can actually act as an ion channel antagonist. Moreover, PMP-072 was clearly distinct from typical competitive antagonists, since it was able to act as a silent agonist. It synergizes with the allosteric modulator PNU-120596, and subsequently activates desensitized α7nAChR. However, PMP-072 was less efficacious than PMP-311 at both channel activation and desensitization, suggesting that both conducting and non-conducting states maybe of importance in driving an anti-inflammatory response. Finally, we found that the anti-arthritic effect can be observed despite limited penetration of the central nervous system.

**Conclusions:**

These data provide direct evidence that the α7nAChR in immune cells does not require typical ion channel activation to exert its antiinflammatory effects.

## Introduction

Rheumatoid arthritis (RA) is a chronic, immune-mediated inflammatory disease of unknown etiology, characterized by nonspecific, often symmetric, inflammation of the peripheral joints. Hallmarks of the disease include inflammation of the synovium leading to destruction of cartilage and bone [1,2]. Although the introduction of anti-tumor necrosis factor (TNF) therapy and other new biologicals has played a major role in improving patient outcomes, RA is still associated with long-term morbidity and early mortality [3] Thus, there is still a need for the identification of new pathways involved in the modulation of inflammation, which could help to increase the efficacy of the RA treatment.

In recent years, it has been demonstrated that the efferent vagus nerve may inhibit inflammatory responses. This process was first described by Tracey and colleagues and has been termed “the cholinergic anti-inflammatory pathway” [4,5]. The key mediator of the cholinergic anti-inflammatory pathway, acetylcholine (ACh), may inhibit pro-inflammatory cytokine release via interaction with members of the nicotinic acetylcholine receptor family (nAChR), and in particular with the α7 subunit (α7 nAChR). This receptor is not only expressed by neuronal cells but also by macrophages and other cells involved in the inflammatory response. In these cells stimulation of the α7 nAChR by ACh or α7-specific agonists suppresses pro-inflammatory cytokine release [4]. Another strategy for activating the cholinergic anti-inflammatory pathway is by vagus nerve stimulation (VNS) using an electrical device. Activation of the cholinergic anti-inflammatory pathway, either by VNS or through pharmacologic approaches, has been shown to significantly ameliorate disease in several animal models, including endotoxemic shock [4,6], septic peritonitis [7], colitis [8], pancreatitis [9], and ischemia-reperfusion injury [10,11].

The cholinergic anti-inflammatory pathway may also be relevant in arthritis. Pharmacological or electrical stimulation of the vagus nerve decreases carrageenan-induced inflammation in the rat paw [12]. Moreover, we have shown that unilateral cervical vagotomy exacerbates collagen-induced arthritis (CIA), whereas treatment with AR-R17779, an α7 nAChR agonist, ameliorates arthritis activity [13]. In addition, α7-deficient mice showed a marked increase in synovial inflammation compared with wild-type littermates [14]. Underscoring the potential importance of α7 nAChR in humans, it has been shown that leukocytes and fibroblast-like synoviocytes (FLS) in the RA synovium express α7 nAChR and α7 nAChR-specific agonists can, *in vitro*, modulate the inflammatory response of RA FLS [15,16].

**Figure 1:**
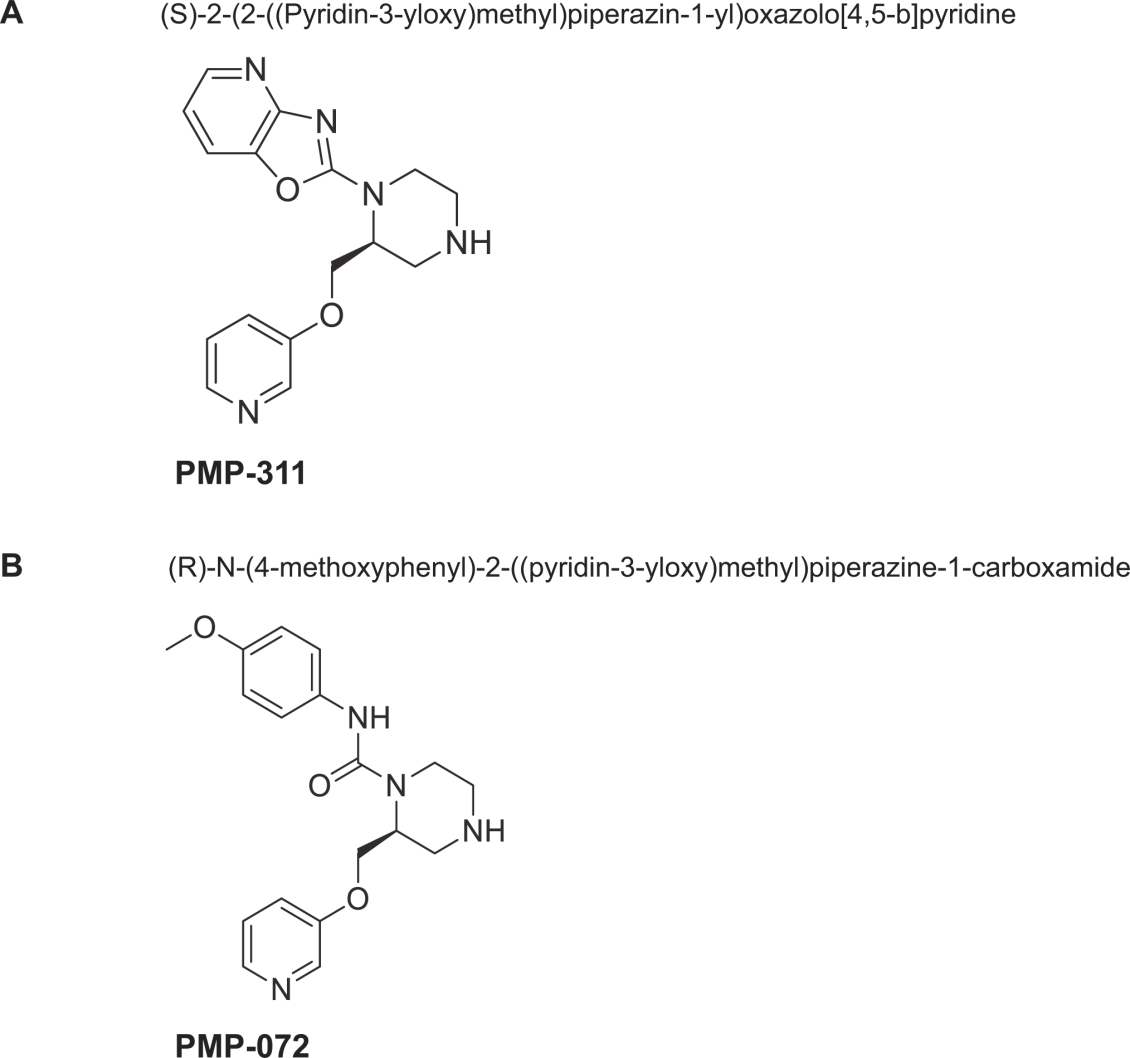
Chemical structures of (A) PMP-311; (*S*)-2-(2-((**P**yridin-3-yloxy)**m**ethyl)**p**iperazin-1-yl)oxazolo[4,5-*b*]pyridine and (B) PMP-072; (R)-N-(4-methoxyphenyl)-2-((**p**yridin-3-yloxy)**m**ethyl)**p**iperazine-1-carboxamide.

The members of the nAChR family form as homopentameric or heteropentameric receptors in neurons, which function as ligand-gated ion channels, and can in the case of the heteropentameric receptors, mediate fast signal transmission at synapses. However, it is at present controversial whether the α7 nAchR in immune cells requires ion channel activity to exert its anti-inflammatory effects. Silent agonists, agents which convert α7 receptors to allosteric modulator sensitive desensitized states without appreciable ion channel activation [17] are a recently described class of agents that may be useful to investigate this hypothesis. Previous studies, have reported that the silent agonist NS6740 [18] is in fact more effective at decreasing a microglia proinflammatory response than were α7 agonists more effective at ion channel activation. In the present study, we describe the binding profile, biological properties, and pharmacological effects of two novel α7nAChR selective small molecules (PMP-311 and PMP-072, Fig. 1) which vary in their ability to activate the α7 ion channel in the presence and absence of the positive allosteric modulator (PAM) PNU-120596, such that PMP-072 only produces significant channel activation when receptors have been modified with the PAM.

## Materials and Methods

### Chemicals

Experimental compounds PMP-311 and PMP-072 were synthesized by Cornerstone Therapeutics, Inc. (Cary, NC) and provided as a hydrochloride or fumerate salt, respectively. The compounds are synthesized as previously described [19,20] and depicted in Fig. 1.

### Binding studies with the rat α7nAChR

Binding studies with rat α7 nAChR were done using the rat pheochromocytoma cell line PC12 that endogenously expresses the α7 nAChR (American Type Culture Collection, Manassas, VA). PC12 cells were maintained in Ham F-12 nutrient mixture, containing 15% horse serum, 2.5% fetal bovine serum (FBS), 2 *mM* L-glutamine, 1.5 g/L NaHCO_3_, 100 units of penicillin, and 100 μg streptomycin.

For the binding assay, PC12 cells were resuspended in binding buffer (phosphate buffered saline with calcium and magnesium, containing 1% FBS and 0.02% NaN_3_) at 1.5 to 2.7 × 10^6^ cells per ml and 55 μl (0.8–1.5 × 10^5^ cells per well) was added to a 96-well, v-bottom plate. Test compounds were diluted in binding buffer, to 2.2 times the desired final concentration, and 55 μl was added to the cells; 55 μl binding buffer was added to the cells in the control wells (total binding, non-specific binding, and cell controls; n = 1–3). Biotinylated α-bungarotoxin (BTx) (Invitrogen) was added to the cells (excluding the cell control) for a final concentration of 10 *nM*. An excess of unlabeled BTx was added to the non-specific binding (NSB) control at a final concentration of 1.5 *μM*. The samples were incubated at room temperature for 1.0 to 1.5 hour(s) and thereafter the cells were washed one time with binding buffer to remove unbound BTx.

Phycoerythrin-labeled streptavidin (streptavidin-PE) (Becton-Dickinson, Franklin Lakes, NJ) was diluted in binding buffer and 50 μl was added to the cells (excluding the cell control) at a 1.0 μg/ml final concentration. The samples were incubated in the dark, at room temperature, for 15 minutes. Thereafter cells were washed one time with binding buffer, to remove the excess streptavidin-PE. The samples were resuspended in 120 μl binding buffer. BTx binding was quantified by fluorescence-activated cell sorting (FACS) analysis. For each concentration of test compound, the displacement of BTx from the α7 nAChR was quantified by measuring the intensity of the fluorescent signal. Raw data units are in percent events (% events), which is equal to the percentage of cells in the total cell population that has a fluorescent intensity greater than the background level. Percent inhibition (% inh) of BTx binding was calculated from the ratio of the % events measured in the sample to the total binding % events, with background (NSB) subtracted:

$$\%\;\mathrm{inh}=\frac{1-(\mathrm{sample}\;\%\;\mathrm{events}-\mathrm{NSB}\;\%\;\mathrm{events})}{\mathrm{total}\;\mathrm{binding}\;\%\;\mathrm{events}-\mathrm{NSB}\;\%\;\mathrm{events}}\times 100$$

Curve-fit analysis was done using GraphPad Prism (GraphPad Software, San Diego, CA), % inh values were plotted versus the log10 of the concentration. Curve-fit analysis was performed using a four-parameter logistic equation:

$$y=\frac{\mathrm{Bottom}+(\mathrm{Top}-\mathrm{Bottom})}{1+10^{\left(\mathrm{LogIC}50-X\right)}}\times \mathrm{Hillslope}$$

with the “Top” parameter constrained at 100% and the “Bottom” parameter constrained at 0%. Ki values were calculated from IC_50_ values using the Cheng-Prusoff equation:

$$Ki=\frac{IC_{50}}{1+\frac{\left[BTx\right]}{KD}}$$

### Broad selectivity panel

Broad selectivity was assessed at Cerep (Celle l’ Evescault, France), as described in the Cerep catalog, though the determination of the effect of PMP-311 and PMP-072 in *in vitro* radioligand receptor binding assays with 52 different receptors, channels, and transporters. The specific ligand binding to the receptors is defined as the difference between the total binding, and the nonspecific binding determined in the presence of an excess of unlabelled ligand. The results are expressed as a percent of control specific binding and as the mean percent inhibition of control specific binding obtained in the presence of 10 *μM* PMP-311 and PMP-072. Individual and mean values are presented in the results section. The IC_50_ values (concentration causing a half-maximal inhibition of control specific binding) and Hill coefficients (*nH*) were determined by non-linear regression as described above.

### Pharmacokinetics

Pharmacokinetic studies were carried out at Cerep using non-cannulated, non-fasted CD1 mice (Charles River Laboratories, Wilmington, MA). PMP-311 or PMP-072 were formulated in phosphate buffered saline (PBS), pH 7.4, as a clear solution and 1 mg/kg was administered by bolus intravenous (IV) injection or 5 mg/kg by oral gavage. Plasma samples were obtained from 3 mice per time point at 15, 30, 60, 120, 240, 360, 480 and 1440 min post-dose. The plasma samples were processed using acetonitrile precipitation and analyzed by HPLC-MS or HPLC-MS/MS to determine the concentration of drug as compared to a plasma calibration curve (aliquots of drug-free plasma were spiked with the test compound at the specified concentration levels and processed together with the unknown plasma samples using the same procedure). The processed plasma samples were stored frozen (−20°C) until the HPLC-MS/MS analysis. Peak areas were recorded, and the concentrations of the test compound in the unknown plasma samples were determined using the respective calibration curve. The reportable linear range of the assay was determined, along with the lower limit of quantitation (LLQ).

Plots of plasma concentration of compound versus time were constructed. The fundamental pharmacokinetic parameters of compound after oral and IV dosing (C_max_: maximum concentration, T_max_: time to maximum concentration, T_1/2_: terminal elimination half-life, AUC: area under the curve, CI: clearance, Vd: volume of distribution, and %F: bioavailability) were obtained from the non-compartmental analysis of the plasma data using WinNonlin (Pharsight, St. Louis, MO).

### cDNA clones and preparation of RNA

Human nAChR receptor clones were obtained from Dr. Jon Lindstrom (University of Pennsylvania, Philadelphia, PA). The RIC-3 clone was obtained from Dr. Millet Treinin (Hebrew University, Jerusalem, Israel). Subsequent to linearization and purification of cloned cDNAs, RNA transcripts were prepared *in vitro* using the appropriate mMessage Machine kit from Ambion Inc. (Austin, TX).

### Receptor expression in *Xenopus* oocytes

The preparation of *Xenopus laevis* oocytes for RNA expression was conducted as previously described [21]. Stage 5 oocytes were isolated and injected with 50 μl (5–20 ng) of each subunit cRNAs. Recordings were conducted 2–5 days after injection. RNA coding for human α7 nAChR was routinely co-injected with the cDNA for human RIC-3, an accessory protein that improves and accelerates α7 nAChR expression without affecting the pharmacological properties of the receptors [22].

### Electrophysiology

Experiments were conducted using OpusXpress6000A (Molecular Devices, Union City, CA) as described before [23]. ACh applications were 12 seconds in duration.

### Experimental protocols and data analysis

Each oocyte received two initial control applications of 300 *μM* ACh, followed by the experimental drug application, and subsequent control application of 300 *μM* ACh, unless otherwise indicated. Responses to experimental drug applications were determined relative to the preceding ACh control responses in order to normalize the data, compensating for the varying levels of channel expression among the oocytes. Responses for α7 nAChR were calculated as net charge [21], since peak currents inaccurately report the agonist concentration dependence of α7 nAChR-mediated responses [24]. For experiments measuring the effects of ACh and the experimental compounds on allosterically modulated receptors, following the acquisition of baseline responses, cells were given a 60 s application of 300 *μM* PNU-120596. We have previously reported that, due to the slow reversibility of PNU-120596’s effects in the oocyte system, this protocol produces modulation which allows previously desensitized receptors to be reactivated and that a significant percentage of the receptors remain in the modulated state for at least 15 minutes [25]. For all experiments, means and standard error of the mean (SEM) were calculated from the normalized responses of at least four oocytes for each experimental concentration, plotted using Kaleidagraph 3.52 (Synergy Software, Reading, PA), and curves were generated from the Hill equation:

$$\mathrm{Response}=\frac{I_{Max}{\left[\mathrm{agonist}\right]}^{n}}{{\left[\mathrm{agonist}\right]}^{n}+{\left(EC_{50}\right)}^{n}}$$

where I_max_ denotes the maximal response for a particular agonist/subunit combination, and n represents the Hill coefficient. I_max_, n, and the EC_50_ were all unconstrained for the fitting procedures except in the case of the ACh concentration-response curves. Because ACh is our reference full agonist, those data were normalized to the observed ACh maximum, and the Imax of the curve fits were constrained to equal 1.

### Animals and ethics

Male DBA/1 mice (8–10 weeks of age) were purchased from Harlan (Horst, The Netherlands). They were housed under specific pathogen-free conditions at the animal facility of the Academic Medical Center, University of Amsterdam. Animals were fed ad libitum. The Institutional Animal Care and Use Committee of the Academic Medical Center approved all experiments. The approval number of the studies is DSK101014 and DSK100689.

### Induction and assessment of collagen-induced arthritis

Collagen-induced arthritis was induced and evaluated as previously described [26–28]. The severity of arthritis was assessed using an established semiquantitative scoring system (0–4; 0 = normal, 1 = swelling in 1 joint, 2 = swelling in >1 joint, 3 = swelling in the entire paw, and 4 = deformity and/or ankylosis [26–28]. The cumulative score for all 4 paws of each mouse was used to represent overall disease severity and progression. Hind paw ankle joint thickness was measured using a caliper For the evaluation of incidence, mice were considered to have arthritis if the arthritis score increased by at least 1 point for 2 or more following days.

### Study design and evaluation of arthritis activity

In study 1, we evaluated the role of the two novel α7nAChR-specific modulators in CIA. The receptors were stimulated by oral gavage of PMP-311 (5 mg/kg; n = 15) or PMP-072 (5 mg/kg; n = 17). The compounds were administered once a day from day 20 until the end of the experiment (day 34). Control mice received saline. In study 2, we evaluated the anti-inflammatory effects of different dosages of the α7nAChR-specific agonists. PMP-311 (2 or 10 mg/kg; n = 15) and PMP-072 (10 or 20 mg/kg; n = 15) were administered in the same way as in study 1. In both studies, mice were inspected daily for signs of arthritis and thickness of hind paws was measured using a caliper from day 16 till sacrifice by 2 independent observers (MAvM and JK) who were not aware of the treatment.

### Radiologic analysis

Hind paws were used for radiographic evaluation. Joint destruction was scored on a scale of 0–4, where 0 = no damage, 1 = demineralization, 2 = 1 or 2 erosions, 3 = severe erosions, and 4 = complete destruction of the joints [13]. The radiographs were scored by 2 independent observers (MAvM and MJV) in a blinded manner; minor differences in scoring between the observers were resolved by mutual agreement.

### Histologic analysis

Hind paws were fixed for 24 hours in 10% buffered formalin and decalcified in 15% EDTA. The paws were then embedded in paraffin, and serial 5 μm sagittal sections of whole hind paws were cut and stained with hematoxylin and eosin (HE). Two independent observers (MAvM and MJV) assessed the tissue for the degree of synovitis by microscopic evaluation, under blinded conditions, as described previously [13,29,30]. Synovitis was graded on a scale of 0 (no inflammation) to 3 (severely inflamed joint) based on the extent of infiltration of inflammatory cells into the synovium.

### Statistical analysis

To evaluate the effects of different treatments, we determined the change in clinical arthritis scores in each mouse from the start of treatment until the end of the experiment. AUC for the change in arthritis scores were calculated. The significance of the differences in the mean changes in scores (clinical, radiologic and histologic) between groups was determined by Kruskal-Wallis test followed by Mann-Whitney U test (SPSS version 12.0.2; SPSS, Chicago, IL). Incidence was compared using Kaplan-Meier survival analysis (GraphPad Prism). *P* values less than 0.05 were considered statistically significant.

## Results

### Functional activity and selectivity of the compounds on α7 nAChR and α4β2 nAChR

**Figure 2:**
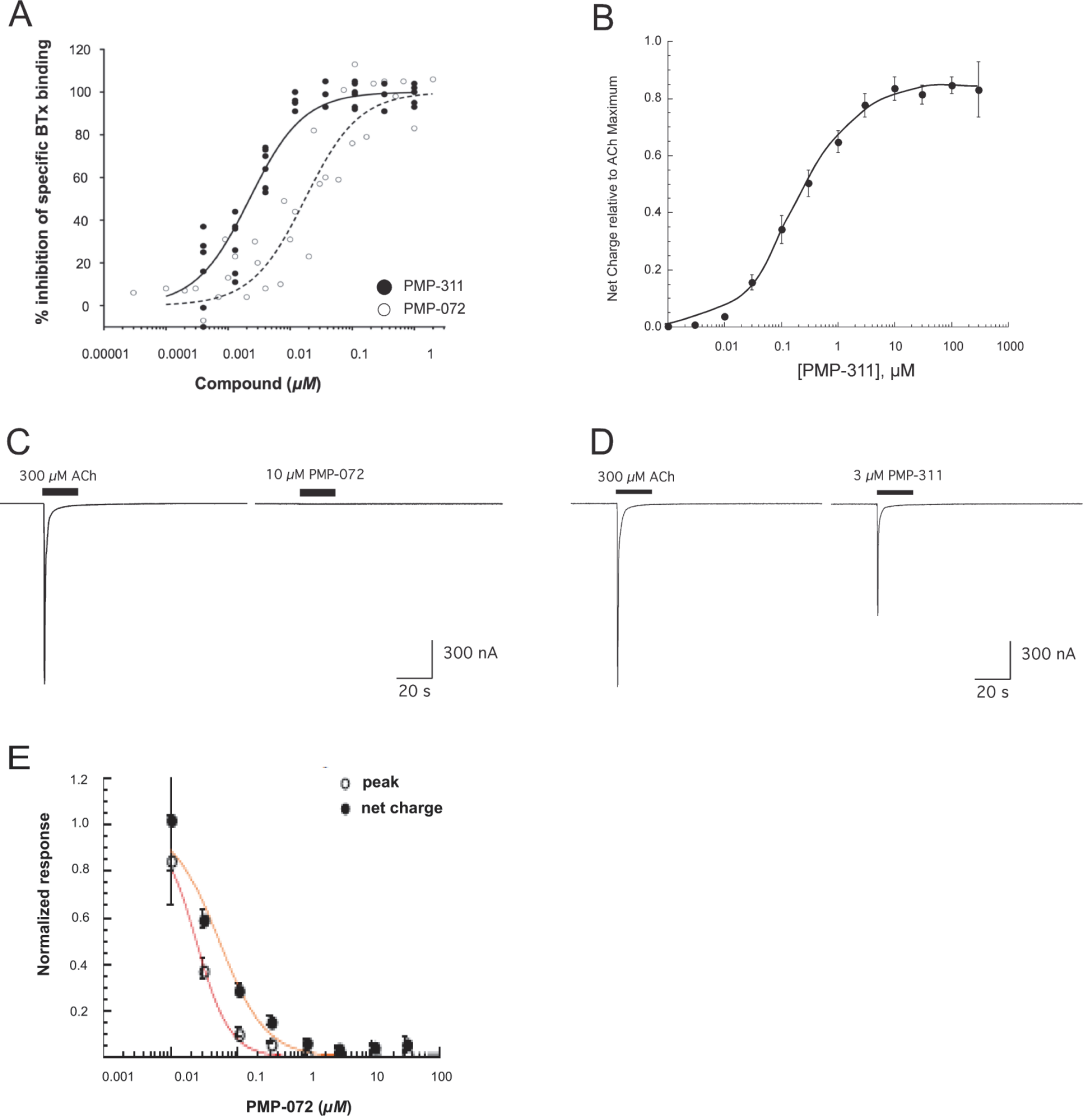
Compound activity of PMP-311 and PMP-072 on α7 nAChR. **A**, Competitive binding assay showed that PMP-311 and PMP-072 displaced the α7 nAChR-specific agonist α-bungarotoxin from binding to PC12 cells. **B**, PMP-311 showed a dose-dependent activation of α7 nAChR with a maximum about 85% that of ACh **C**, PMP-072 does not show to be an agonist of α7 nAChR ion channel activity. **D**, Voltage-clamp electrophysiological techniques revealed that application of PMP-311 (3 *μM)* to *Xenopus* oocytes expressing human α7 nAChR elicited a typical inward current. **E**, When PMP-072 was co-applied with 60 μM ACh to Xenopus oocytes voltage-clamped at 60 mV there was a concentration dependent decrease in the responses compared to ACh applied alone suggesting that with this experimental approach PMP-072 an antagonist of ACh-evoked α7 nAChR channel activation. In panels B & E each point represents the average of at least four cells (±SEM). Data were normalized to control responses to 300 μM ACh obtained prior to the application of PMP-311 (panel B) or 60 μM ACh applied without PMP-072 (panel E).

In a competitive binding assay, compounds PMP-311 and PMP-072 displaced the α7 nAChR-specific agonist α-bungarotoxin from binding to cultured PC12 cells (derived from a pheochromocytoma of rat adrenal medulla) that endogenously express the α7-subunit. Both compounds showed potent binding to α7 nAChR with Ki values of 0.9 *nM* or 6.9 *nM*, respectively (Table 1, Fig. 2A). PMP-311 has some affinity for α4β2 nAChR (cytosine binding to rat brain membranes; Ki = 30 *nM*). Both PMP-311 and PMP-072 do not exhibit any affinity for the muscle receptor (α-bungarotoxin binding to TE671 cell membranes; Ki > 100,000 *nM*) (Table 1).

**Table 1:** Binding affinities of PMP-311 and PMP-072 for α7nAChR, α4β2 nAChR and α1β1 nAChR.

**nAChR**	**PMP-311**	**PMP-072**
α7nAChR^1^	0.9 ± 0.2 *nM*	6.9 ± 1.4 *nM*
α4β2nAChR^2^	30 *nM*	≥100,000 *nM*
α1β1nAChR^3^	>100,000 *nM*	>100,000 *nM*

^1^a-btx binding to rat PC12 cell membranes

^2^cytisine-binding to rat brain membranes

^3^a-btx binding to TE671 cell membranes

In addition, the binding selectivity of both compounds with respect to other nAChRs, as well as to a broader selection of targets was evaluated by testing for competition in radioligand binding assays with 52 pharmacologically important receptors, channels, and transporters. PMP-311 shows very little interaction with the panel of targets, with the exception of the human serotonin transporter (72% inhibition at 10 *μM*). PMP-072 also does not show any significant interactions with any of these additional targets with the exception of the human serotonin transporter (Ki = 1800 *nM*). This includes the α4β2nAChR (cytosine binding to rat brain membranes; Ki > 100,000 *nM*), and muscle receptor (α-bungarotoxin binding to TE57 cell membranes; Ki > 100,000 *nM*) (data not shown).

Using voltage-clamp electrophysiological techniques, we examined the functional activity of both compounds at human α7 nAChR, expressed in *Xenopus* oocytes in comparison with ACh responses. It is known that the maximal channel activation, measured as net charge, is achieved with the application of 300 *μM* ACh and that application of concentrations greater than 300 *μM* produce no further increase in response [31]. Application of 3 *μM* PMP-311 to *Xenopus* oocytes elicited a typical inward current (Fig. 2D), indicative of α7 nAChR agonist activity. The maximum responses to PMP-311 were about 85% compared to ACh with an EC_50_ of about 200 *nM* (Fig. 2B). Compound PMP-072 did not appear to be an agonist of the α7 nAChR ion channel activity in the *Xenopus* oocyte membrane current assay (Fig. 2C) under control conditions, but by virtue of its binding to α7 nAChR it could act as an antagonist of ACh-stimulated α7 nAChR channel activity, with an IC_50_ of 20–50 *nM* (Fig. 2E).

Both compounds were also tested for potential effects on α4β2 nAChR. As observed in the competitive binding assays PMP-311 does interact with α4β2 nAChR; in the ion channel assays it was shown to be a potent inhibitor of α4β2 nAChR with an IC_50_ below 1 *μM* (data not shown). The mechanism of inhibition is probably related to competition.

### Pharmacokinetics

The pharmacokinetic properties of both compounds in mouse are shown in Table 2. Following an oral dose of 5 mg/kg maximum plasma concentrations (C_max_) were 2.5 *μM* (787 ng/ml) at 15 min and 0.94 *μM* (324 ng/ml) at 30 min for PMP-311 and PMP-072, respectively. The bioavailability (%F), used to describe the fraction of the orally administered dose of unchanged compound that reaches the systemic circulation, was 50% for PMP-311 and 76% for PMP-072. Moreover, both compounds had comparable relatively short plasma half-lives in mice. Brain penetration was measured 30 minutes after intravenous administration of the compounds. PMP-311 showed 44% brain penetration whereas PMP-072 only showed 6% of brain penetration.

**Table 2:** Compound pharmacokinetics in mice.

		**PMP-311**	**PMP-072**
**5 mg/kg oral**	C**_max_**	787 ng/ml (2.5 *μM*)	324 ng/ml (0.94 *μM*)
	T_max_	15 min	30 min
	T_1/2_	98 min	104 min
	AUC	34033 min ng/mL	35259 min ng/mL
	%F	50%	76%
**1 mg/kg IV**	T_1/2_	19 min	39 min
	Cl	72 mL/min/kg	103 mL/min/kg
	Vd	1936 mL/kg	5833 mL/kg
	Brain penetration*	44%	6%

* relative to plasma level 30 min after administration of 1 mg/kg IV

### Stimulation of the α7nAChR ameliorates arthritis activity and reduces disease incidence

**Figure 3:**
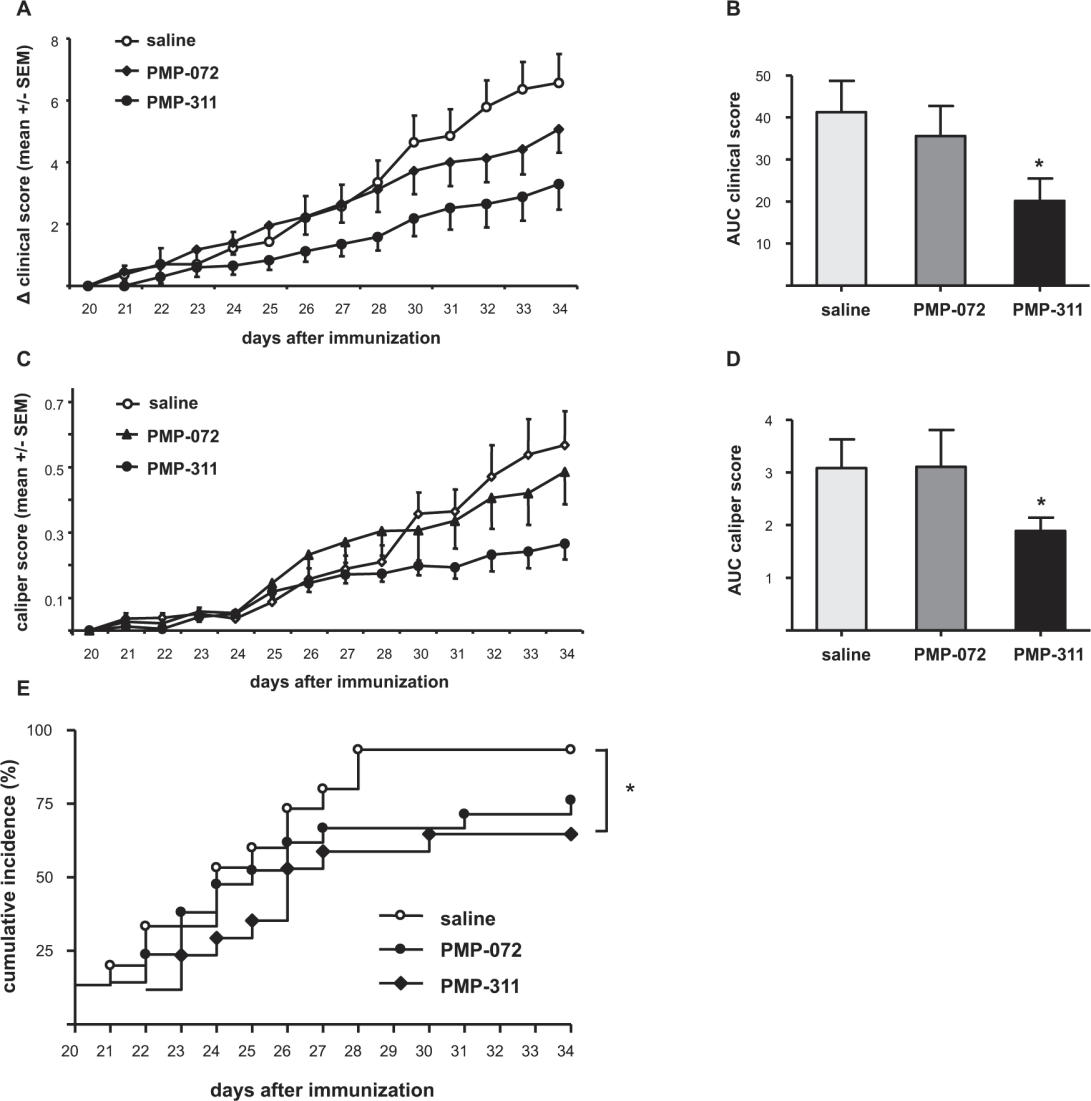
Treatment with PMP-311 resulted in an amelioration of clinical signs of arthritis. Arthritis was induced in mice by immunization with type II collagen, and mice were treated with PMP-311 (n = 15, 5 mg/kg), PMP-072 (n = 17, 5 mg/kg), or saline (n = 15) by oral gavage from day 20 until day 34. **A**, Clinical score; Mice treated with PMP-311 showed a decrease in arthritis scores compared to saline-treated mice. **B**, Area under the curve (AUC) of the clinical score (day 20 to day 34) was decreased in PMP-311-treated mice versus control mice. **C**, Caliper score; Mice treated with PMP-311 showed a decrease in hind paw thickness, measured daily with a caliper, compared to the control group. **D**, AUC of the caliper score was decreased in PMP-311-treated mice compared with saline-treated mice. **E**, Disease incidence; PMP-311 reduced the incidence and delayed the onset of arthritis. * *P* < 0.05. compared to the control group.

Mice were treated with PMP-311 or PMP-072 at 5 mg/kg. Both compounds were administered daily by oral gavage from day 20 until day 34 and all mice tolerated the drug treatment well. Control mice received saline. Treatment with PMP-311resulted in an amelioration of clinical signs of arthritis when clinical scores of all treated animals, with and without arthritis, are included in the figure (Fig. 3A). The AUC was decreased by 51% (*P* < 0.05) in mice treated with PMP-311 compared to control mice (Fig. 2B). This effect was accompanied by a decrease in paw swelling in the mice treated with PMP-311 showing a decrease of 39% compared to saline-treated mice (*P* < 0.05) (Fig. 3C and D). Moreover, treatment with PMP-311 resulted in reduced disease incidence and delayed onset of disease (*P* < 0.05) (Fig. 3E). As six out of 15 animals did not develop arthritis the reduction in clinical score at a dose of 5 mg/kg is mainly caused by the animals not developing arthritis. PMP-072 did not significantly ameliorate arthritis activity but a trend towards reduced incidence of disease was seen (Fig. 3A-E).

### PMP-311 treatment reduces bone degradation and synovial inflammation in knee joints

**Figure 4:**
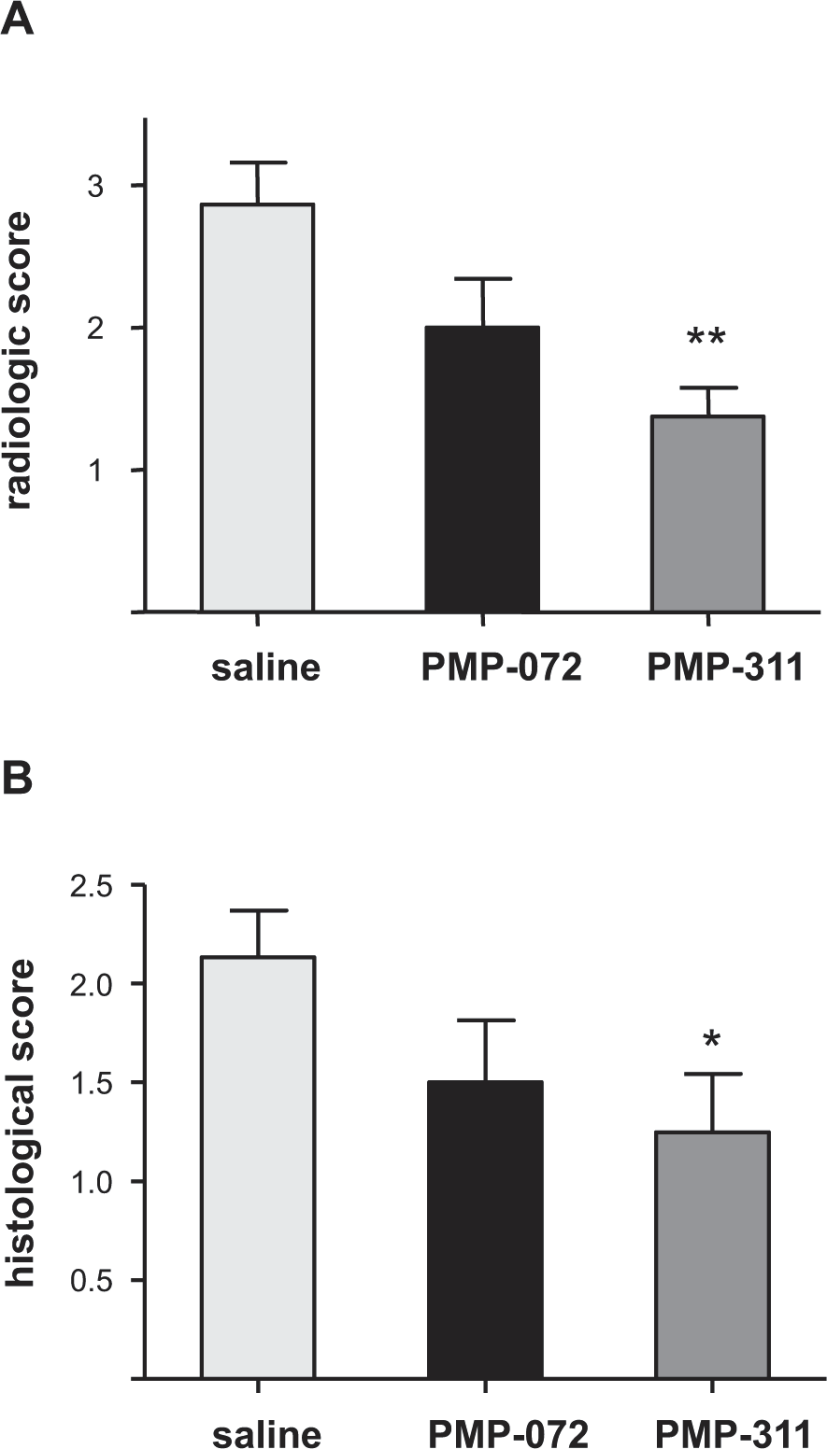
Inhibition of bone degradation and reduction of synovial inflammation in murine collagen-induced arthritis by PMP-311 (n = 15, 5 mg/kg). **A**, Semiquantitative scores of joint destruction. Joint destruction was decreased in mice treated with PMP-311. ** *P* < 0.01 compared to saline-treated mice **B**, Semiquantitative scores for synovial inflammation, assessed by hematoxylin and eosin staining of the knee joints, showed a decrease of synovitis in PMP-311-treated mice. * *P* < 0.05 compared to the control group.

To examine the effects of α7 nAChR-specific ligands PMP-311 (5 mg/kg) and PMP-072 (5 mg/kg) on bone degradation, radiographs of knee joints collected at the end of the experiment were evaluated. Consistent with the effect on arthritis activity, mice treated with PMP-311 showed a significant reduction in joint destruction compared with saline-treated mice (*P* < 0.01), whereas PMP-072 did not reduce bone degradation (Fig. 4A). Similarly, there was a significant reduction of synovial inflammation, assessed by HE staining of knee joints, in mice treated with PMP-311 (*P* < 0.05) (Fig. 4B).

### Dose-response study of the effects of PMP-311 and PMP-072 on arthritis scores and paw swelling

**Figure 5:**
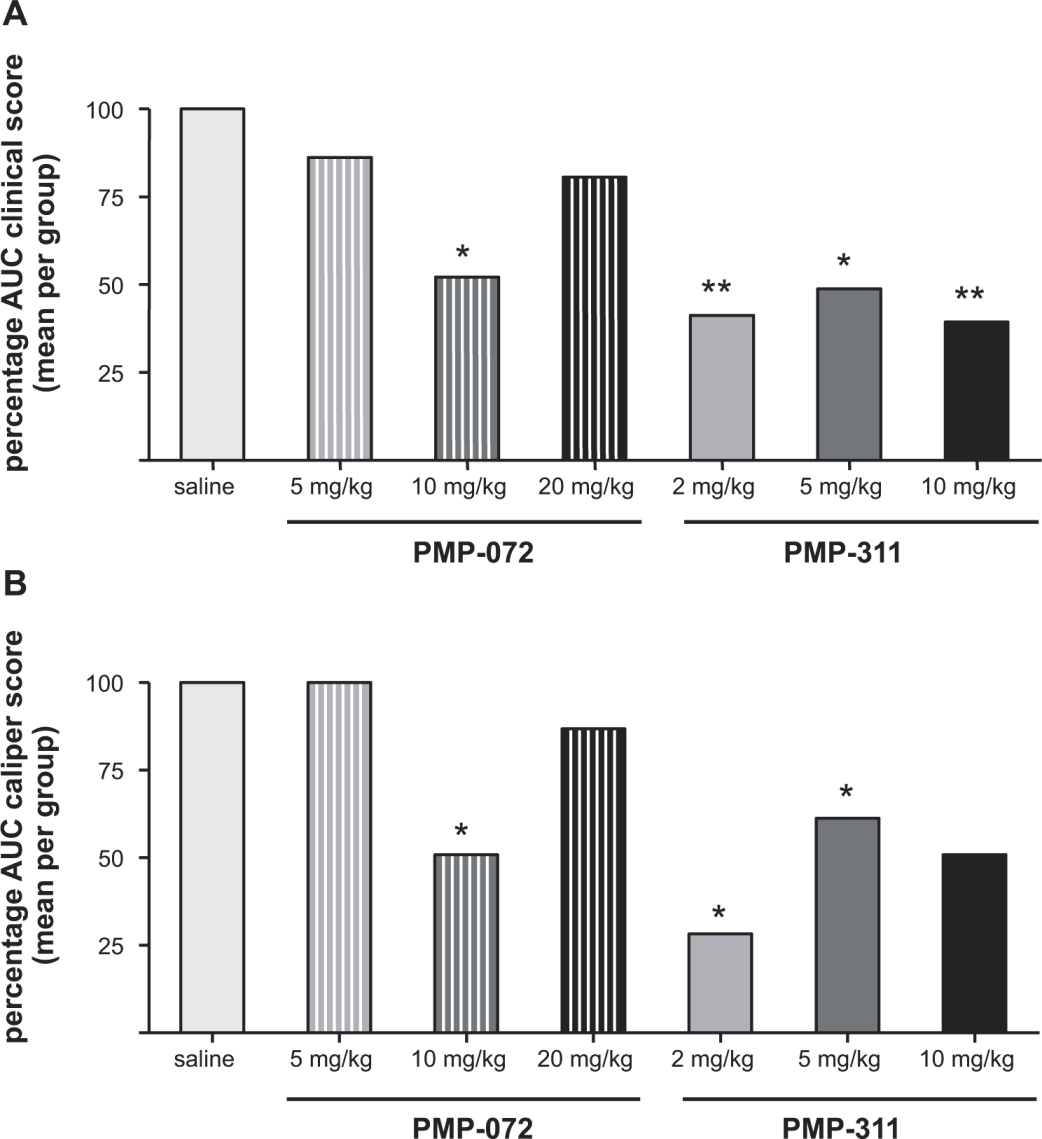
Dose-response study of PMP-311 and PMP-072 on clinical arthritis and paw swelling in murine collagen-induced arthritis. Mice were treated daily with PMP-311 (2, 5, 10 mg/kg, n = 15), PMP-072 (5, 10, 20 mg/kg, n = 15) or saline (n = 15) by oral gavage from day 20 until day 34. Percentages of areas under the curve (AUC) are shown. **A**, AUC of the clinical score was decreased more pronounced in PMP-311-treated mice versus control mice than in mice treated with PMP-072. **B**, Paw swelling was decreased in mice treated with PMP-311 at doses of 2 and 5 mg/kg and in mice treated with PMP-072 at 10 mg/kg. * *P* < 0.05 and ** *P* < 0.01 compared to saline treated mice.

**Figure 6:**
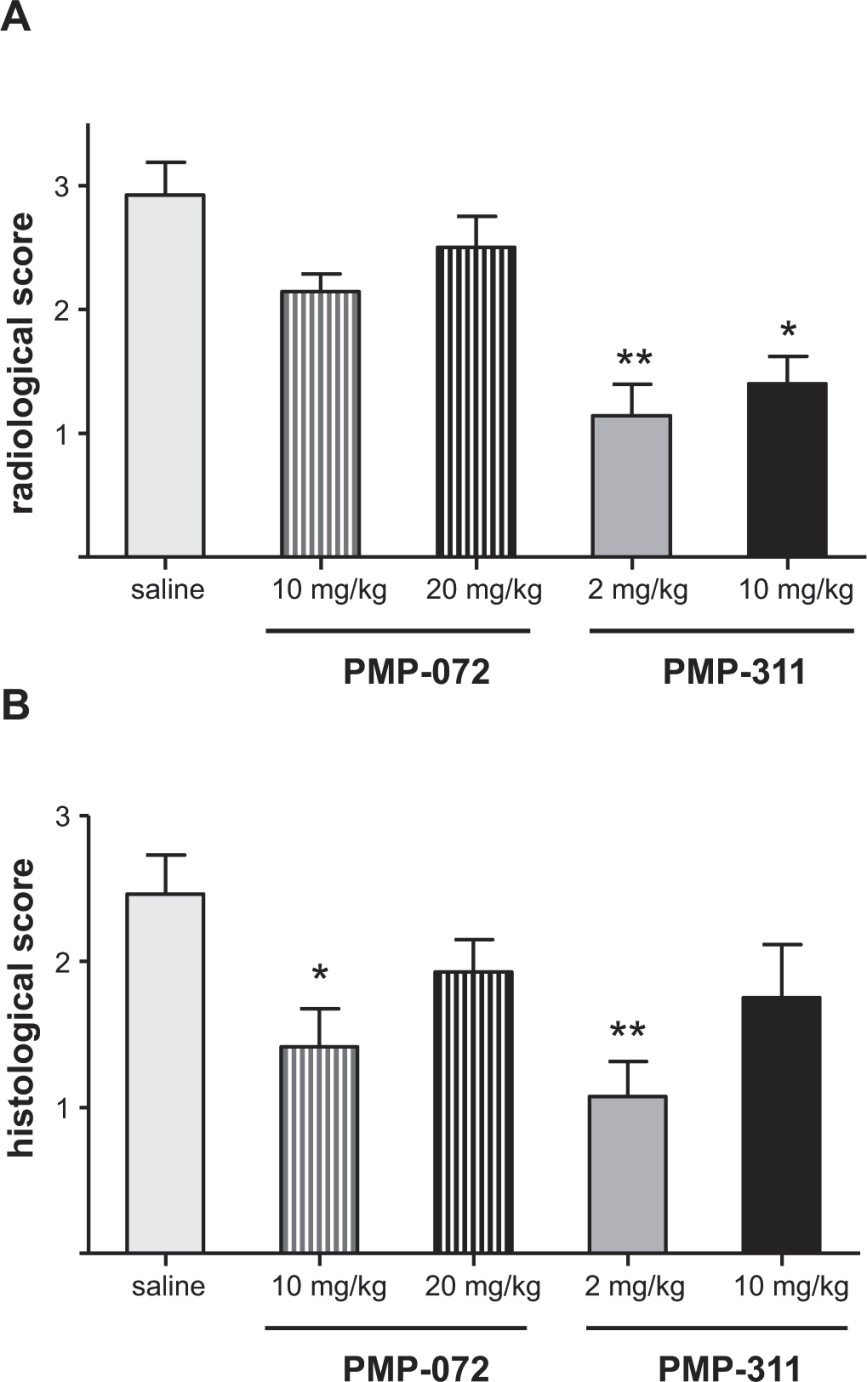
Inhibition of bone degradation and reduction of synovial inflammation in murine collagen-induced arthritis at different doses of PMP-311 (n = 15) and PMP-072 (n = 15) given by oral gavage from day 20 until day 34. **A**, Semiquantitative scores for radiographic joint destruction of the knee joints. Joint destruction was significantly decreased in mice treated with PMP-311 2 mg/kg and 10 mg/kg compared to the control group. **B**, Semiquantitative scores for synovial inflammation, assessed by hematoxylin and eosin staining of the knee joints, showed a decrease of synovitis in mice treated with PMP-311 2 mg/kg and PMP-072 10 mg/kg. * *P* < 0.05 and ** *P* < 0.01 versus saline-treated mice.

Having shown proof of concept that PMP-311 treatment results in decreased arthritis activity, we next performed an independent dose-response study in mice with CIA. We tested the effects of PMP-311 in 1 lower and 1 higher dosage than used in study 1: 2 mg/kg and 10 mg/kg. Because PMP-072 showed a trend towards amelioration of clinical arthritis but was less potent in the receptor studies, we tested in the same experiment the effects of 2 higher doses: 10 mg/kg and 20 mg/kg. All of the animals tolerated the drug treatments well. To allow comparison with the results obtained in study 1, we calculated the percentages of the score compared to the control group. This experiment confirmed the beneficial effect of treatment with 5 mg/kg PMP-311 (a reduction of 49% in clinical score compared to saline (*P* < 0.05) (Fig. 5A)). Arthritis scores were also significantly lower after treatment with PMP-311 at either 2 mg/kg or 10 mg/kg with a reduction of 40% and 39%, respectively, compared to saline-treated mice (*P* < 0.01) (Fig. 5A). In the study where PMP-311 was administered at a dose of 2 mg/kg, there is a clear reduction of clinical score in the arthritic mice (p = 0.032). This decrease was mainly due to a decrease in inflammation since 14 out of 15 mice developed arthritis, These results suggest that all dosages were in the therapeutic range. The most pronounced effect of treatment on paw swelling was observed after low dose treatment (Fig. 5B). Of importance, treatment with PMP-311 2 mg/kg also resulted in a significant reduction in joint destruction (Fig. 6A) and synovial inflammation (Fig. 6B) compared with saline-treated mice (*P* < 0.01).

We also confirmed a trend towards improvement after treatment with 5 mg/kg PMP-072. Of importance, there was a reduction of 48% in arthritis scores compared to saline-treated mice after treatment with 10 mg/kg PMP-072 (*P* < 0.05), but there was no improvement with the higher dosage of 20 mg/kg (Fig. 5A). The beneficial effect of 10 mg/kg PMP-072 was also shown by a decrease in paw swelling (*P* < 0.05) (Fig. 5B). In line with these clinical effects, PMP-072 10 mg/kg treatment resulted in significantly lower scores for synovitis and a trend towards reduced joint destruction compared to saline-treated mice (Fig. 6A and B). The dosages needed to achieve a clinical effect were higher for PMP-072 compared to PMP-311, which is consistent with differences in pharmacokinetics and in binding to the α7 nAChR. In addition, the highest dosages appeared to be less effective than lower dosages, perhaps related to desensitization and loss of biologic response of the receptor due to sustained agonist stimulation.

### Effects of PMP-311 and PMP-072 on α7 nAChR primed with the positive allosteric modulator PNU-120596

**Figure 7:**
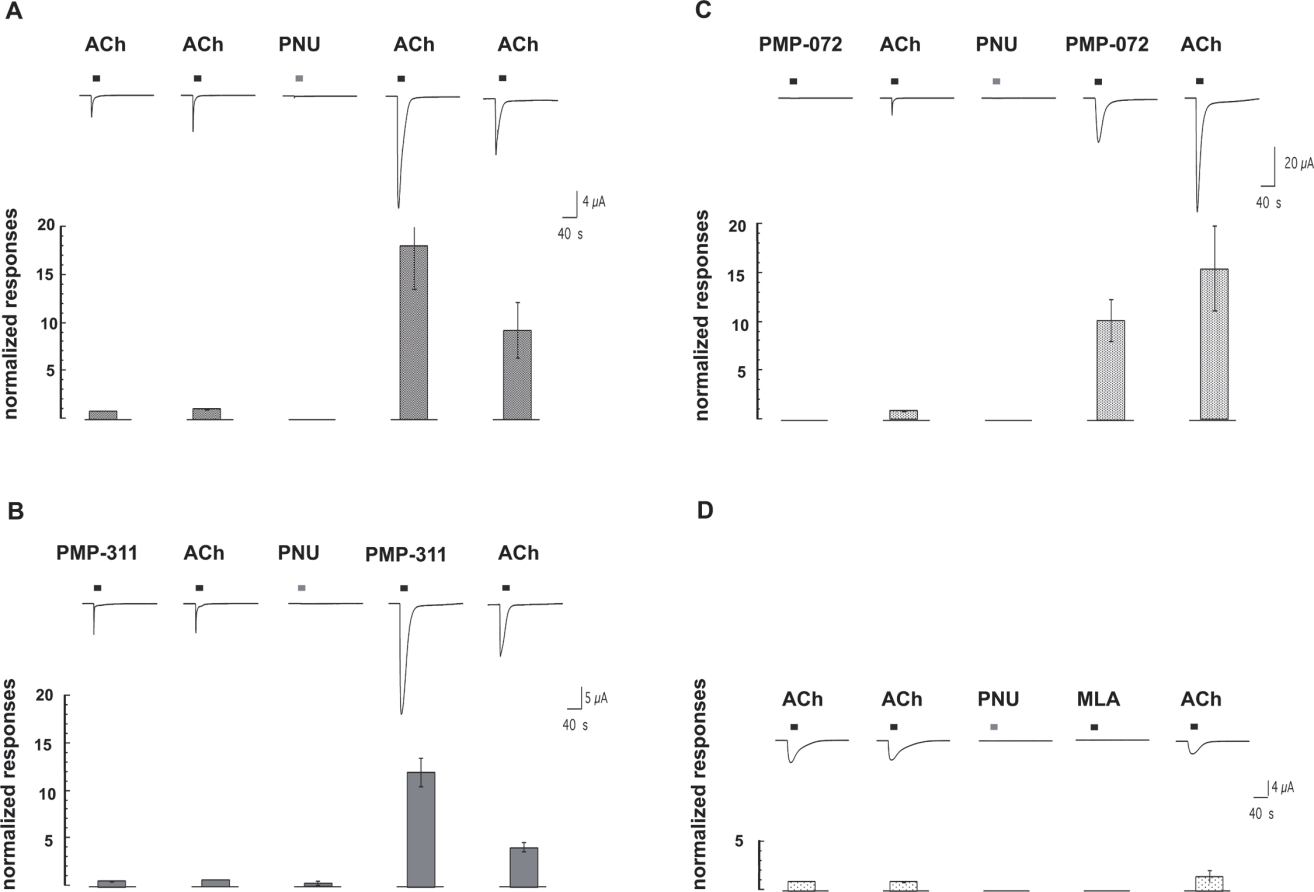
Differential effects of PMP-311 and PMP-072 on human α7 nAChR in the resting state and following priming with the positive allosteric modulator PNU-120596. **A**, Initial responses to applications of 60 *μM* ACh (indicated by the bars) were obtained and then the α7-expressing cells were given a priming application of 300 *μM* PNU-120596 for 60 s (indicated by the bars over the third trace). No ion channel current was stimulated during the PNU-120596 application but subsequent responses to ACh were greatly increased in amplitude and duration. Note that 60 *μM* ACh was used for the control responses in this experiment since the effects of PNU-120596 priming on 300 *μM* ACh-evoked responses often resulted in responses that were too large to record under voltage-clamp conditions. **B**, Initial responses of α7 nAChR-expressing cells to applications of 60 *μM* ACh and 1 *μM* PMP-311 before and after priming with PNU-120596 (third trace). **C**, Initial responses of α7 nAChR-expressing cells to applications of 60 *μM* ACh and 10 *μM* PMP-072 before and after priming with PNU-120596 (third trace). **D**, Initial responses of α7 nAChR-expressing cells to applications of 60 *μM* ACh and the effect of an application of *10 μM* methyllycaconitine (MLA) after priming with PNU-120596. In each panel five sequential 210 intervals of data are shown which were separated by 30s of additional wash (not shown). The bar graphs in each panel represent the average peak current responses of at least four oocytes (±SEM), normalized to the peak current of an initial *60 μM* ACh-evoked response.

PNU-120596 is an α7 nAChR-selective type 2 PAM [32,33] that among other effects can convert desensitized receptors into a conducting state and impede the reversion of receptors back to the PAM insensitive desensitized state(s). Since PNU-120596 itself is not an agonist, the effects of PNU-120596 on the reactivation of desensitized receptors requires either the co-application of PNU-120596 with a desensitizing drug, or the priming of the receptors with an application of PNU-120596 which when applied alone produces no ion channel activation. In the oocyte system, the priming effect of a PNU-120596 applied at a high concentration persists for more than 15 minutes [34]. The enhancement of ACh evoked responses by PNU-120596 priming is shown in Fig. 7A. ACh-evoked responses are increased both in amplitude and duration, since desensitized states are destabilized and conversions to novel conduction states occur [35,36]. As shown in Fig. 7B, responses of PNU-120596 primed cells to PMP-311, which under normal conditions functions as an apparent α7 nAChR agonist, are similar to the responses of primed cells to ACh. In contrast (Fig. 7C), PMP-072, which does not produce detectable ion channel activation under normal conditions, is nonetheless able to activate large ion channel currents in PNU-120596 primed cells. This result suggests that although PMP-072 is able to inhibit ACh-evoked responses in co-application experiments, it may not be a true antagonist, but rather, an α7 nAChR-selective silent agonist [17,37]. To confirm that true competitive α7 nAChR antagonists do not produce ion channel currents in PNU-120596 primed cells, we applied the widely-used α7 nAChR-selective competitive antagonist methyllycaconitine (MLA) to PNU-120596 primed cells [38]. As shown in Fig. 7D, not only did MLA fail to activate the primed cells, the MLA application had residual effects, inhibiting the potentiating of a subsequent ACh-evoked response.

## Discussion

The identification of α7 nAChR as a potential therapeutic target for several diseases, including RA [39], has stimulated the development of α7 nAChR-selective drugs [40]. The present study investigated the pharmacological properties of 2 novel α7 nAChR-specific compounds (PMP-311 and PMP-072) with high oral bioavailability in the mouse. In addition, we tested their therapeutic potential in the CIA model of RA.

Both compounds reduced the clinical arthritis score in CIA by reducing the inflammation and preventing onset of disease. The dosages needed to induce improvement of arthritis are higher for PMP-072, which is expected based on differences in binding to the α7 nAChR. In spite of its relatively high affinity for the α7 nAChR, PMP-072 produced negligible ion channel activation. Molecules like PMP-072 are examples of silent agonists [17], which are compounds with low ion channel efficacy, but can still be single transducers and channel activators in combination with positive allosteric modulator, such as PNU-120596. The prevailing hypotheses for how α7 nAChR mediate the sorts of downstream signal transduction pathways that regulate chemokine release and effects are based on the assumption that the α7-mediated ion currents (in particular the calcium ion component of the currents) provide the crucial initiating step for all downstream effects. With this model, the low efficacy of PMP-072 to stimulate ion channel current would be consistent with a lack of anti-inflammatory activity. However, PMP-072 had an anti-inflammatory effect in CIA at concentrations of 10 mg/kg, consistent with the hypothesis that α7 nAChR ion channel activity may not always be required for α7-mediated signal transduction that leads to down modulation of inflammation, although we cannot eliminated the possibility that it has some unknown off-target activity. Even under the most optimal conditions the steady state P_open_ of α7 nAChR is very low (less than 10^−6^, [41]), and there are many examples where it has been shown that α7 nAChR activate signal transduction pathways not associated with ion channel currents in non-neural cells [5,42–45]. The pathways shown to be potentially activated by α7 nAChR include Jak-STAT and NFκB [42–44], Toll receptor-mediated signaling [46], Bac-Bcl [47], HMGB1-TNF [45], phospholipase C/IP3 [48], and the Ras/Raf-1/MEK1/ERK pathway [42,49]. In many cases, although clearly dependent on the presence of α7 nAChR, and putative agonists, the activation of the signal transduction mechanisms appear to be independent of α7 nAChR ion channel activation [46,48]. These observations support the hypotheses that α7 nAChR may function in multiple ways and suggest that various ligands may differ in their ability to stimulate ion channel activation and/or signal transductionAlternatively, the forms of α7-type receptors expressed in the non-neuronal cells which mediate anti-inflammatory cholinergic effects may be intrinsically different from the ion-channel forms of α7 nAChR that are expressed in neurons [50]. Future studies will have to be performed to elucidate the exact mechanism of action of PMP-311 and PMP-072.

We hypothesize that, although PMP-072 is functionally an antagonist of α7 nAChR ACh-evoked ion channel activation, it is nonetheless an agonist for ion channel-independent signal transduction. Another silent agonist, NS-6740 has been shown to reduce LPS-induced TNF release in microglia [18], but it was unable to improve memory retention in a cognitive mouse model [51]. The α7 nAChR-selective partial agonist GTS-21 (DMXBA) is also relatively ineffective at activating the α7-receptor’s ion channel and yet has been shown to be very effective in several models for suppressing peripheral inflammation [6,9,52–54]. We have shown that a factor limiting the efficacy of GTS-21 is its tendency to preferentially induce a stable desensitized state of the receptor, an effect that can be revealed with the type 2 positive allosteric modulator PNU-120596 [34]. We have hypothesized that the state in which the ion channel is desensitized may nonetheless be an active mediator of signal transduction. In this work we show that although PMP-072 is ineffective at activating α7 nAChR-mediated ion currents, it does modulate the expression of PNU-120596-sensitive desensitization.

In addition to differences in affecting ion channel activation, there were also other differential effects between PMP-311 and PMP-072. Binding studies showed that PMP-311 is quite selective and had high affinity for rat α7 nAChR, whereas it showed lower affinity for the other nAChR tested. Functional electrophysiological experiments using human nAChR expressed in *Xenopus* oocytes confirmed that when PMP-311 binds to α7 nAChR, it functions as a conventional agonist, whereas its binding to other nAChR subtypes does not produce ion channel activation. Specifically, PMP-311 acted as an antagonist of the α4β2 nAChR (IC_50_ ≈ 20 *nM*, data not shown). PMP-072 had a lower affinity for rat α7 nAChR than PMP-311, but it was more selective than PMP-311 in binding to α7 nAChR relative to α4β2nAChR. PMP-311 showed the ability to inhibit the serotonin transporter with 72% at a concentration of 10 μM. Inhibition of the serotonin transporter will increase serotonin availability, which could potentially lower inflammation [55], however levels of 10 μM were not reached in the animal studies. Of note, the previously described α7-selective agonist AR-R17779 also showed an anti-inflammatory effect in CIA [13]; the fact that AR-R17779 selectively activates α7 nAChR without significant antagonism of α4β2 nAChR [56], suggests that α4β2 nAChR antagonist activity of PMP-311 is not required for its efficacy in treating of CIA. This notion is supported by the anti-inflammatory effect of PMP-072 described here, since it is less effective in binding to α4β2 nAChR than PMP-311. Finally, PMP-072 exhibited markedly lower brain penetration than PMP-311.

## Conclusions

Collectively, the results of this study confirm and extend previous work showing that α7 nAChR ligands may reduce arthritis activity, prevent onset of disease and protect against joint destruction in the CIA model of RA. Of importance, we provide direct evidence that α7nAChR agonists may exert their anti-inflammatory effect independent of ion channel activation.
